# Single-Atom Nanozymes Linked Immunosorbent Assay for Sensitive Detection of A*β* 1-40: A Biomarker of Alzheimer's Disease

**DOI:** 10.34133/2020/4724505

**Published:** 2020-10-19

**Authors:** Zhaoyuan Lyu, Shichao Ding, Nan Zhang, Yang Zhou, Nan Cheng, Maoyu Wang, Mingjie Xu, Zhenxing Feng, Xiangheng Niu, Yuan Cheng, Chao Zhang, Dan Du, Yuehe Lin

**Affiliations:** ^1^School of Mechanical and Materials Engineering, Washington State University, Pullman, WA 99164, USA; ^2^Institute of High Performance Computing, Institute of High Performance Computing, Singapore 138632; ^3^School of Chemical, Biological, and Environmental Engineering, Oregon State University, Corvallis, OR 97331, USA; ^4^Irvine Materials Research Institute (IMRI), University of California, Irvine, CA 92697, USA; ^5^Department of Electrical and Computer Engineering, National University of Singapore, Engineering Drive 3, Singapore 117583

## Abstract

Single-atom nanozymes (SANs) possess unique features of maximum atomic utilization and present highly assembled enzyme-like structure and remarkable enzyme-like activity. By introducing SANs into immunoassay, limitations of ELISA such as low stability of horseradish peroxidase (HRP) can be well addressed, thereby improving the performance of the immunoassays. In this work, we have developed novel Fe-N-C single-atom nanozymes (Fe-N_x_ SANs) derived from Fe-doped polypyrrole (PPy) nanotube and substituted the enzymes in ELISA kit for enhancing the detection sensitivity of amyloid beta 1-40. Results indicate that the Fe-N_x_ SANs contain high density of single-atom active sites and comparable enzyme-like properties as HRP, owing to the maximized utilization of Fe atoms and their abundant active sites, which could mimic natural metalloproteases structures. Further designed SAN-linked immunosorbent assay (SAN-LISA) demonstrates the ultralow limit of detection (LOD) of 0.88 pg/mL, much more sensitive than that of commercial ELISA (9.98 pg/mL). The results confirm that the Fe-N_x_ SANs can serve as a satisfactory replacement of enzyme labels, which show great potential as an ultrasensitive colorimetric immunoassay.

## 1. Introduction

Single-atom catalysts (SACs), which contain exclusively isolated metal active sites, have attracted vast attention due to the precise design of nanomaterials at atomic levels [1–3]. Separated metal atoms enable SACs with remarkable catalytic activity and gratifying stability due to their much higher surface energy, maximum metallic atom utilization, homogeneity of active sites, and particular geometric structure [4–6]. Thereinto, SACs with enzyme-like characteristics are called single-atom nanozymes (SANs) and are regarded as ideal candidates to mimic the structure and catalytic activity of natural enzymes [7–10]. Nowadays, the SANs have found their extensive application as substitutions for natural enzymes in immunoassays [11, 12], environmental treatment [13], biodetection, and biosensing [8, 14, 15] owing to their excellent performance, high stability, ease of large-scale production, and economical price.

Commercial enzyme-linked immunosorbent assay (ELISA) has been a widely recognized standard in food safety, clinical diagnosis, and environmental evaluation due to its relatively high specificity and accuracy [16–19]. Its mechanism is to convert the interactions between antigen and antibody into visible color change so one can easily obtain the results from observation. However, there are still some limitations in the accurate detection of diseases utilizing a commercial ELISA, because the concentration of biomarkers is usually ultralow in the early stages of diseases and the performance of HRP used in ELISA is highly dependent on pH and temperature [20–22]. As such, it is urgent to seek a suitable substitution for natural peroxidase HRP and place continuous efforts on enhancing the sensitivity of ELISA. As we mentioned above, various kinds of SANs possess high enzyme-like characteristics and integrating them into traditional ELISA may enhance the detection performance of immunosorbent assay [15, 23].

Amyloid beta 1-40 (A*β* 1-40), as one of the most plentiful substances in humans, will easily form insoluble toxic A*β* 1-40 aggregation, which is a vital neuropathological hallmark to identify Alzheimer's disease commonly; the clinically relevant range of A*β* 1-40 is several ten to several hundred pg/mL [24, 25]. It is well known that AD begins in the human body years before symptoms present, so detecting A*β* 1-40 at low concentration is of great importance and can be used to estimate the risk or show the presence of AD at early stage [26–28]. Hence, we designed novel high-density Fe-N_x_ single-atom peroxidase-like nanozymes (Fe-N_x_ SANs) from pyrolyzed polypyrrole (PPy) nanotube via a nanoconfined strategy. A series of analyses revealed their ultrahigh surface area and superior peroxidase-like activity. The peroxidase-like catalytic activity of the Fe-N_x_ SANs was optimized and compared with natural HRP, which showed better thermal and pH stable catalytic properties. Then, the streptavidin- (SA-) functionalized Fe-N_x_ SANs were used to replace HRP in ELISA and detect A*β* 1-40. The detection performance of the proposed Fe-N_x_ SAN-linked immunosorbent assay (SAN-LISA) was examined and compared with commercial ELISA. The results show that the SAN-LISA exhibited higher sensitivity, making Fe-N_x_ SANs qualify as an enzyme replacement and providing satisfactory feasibility in clinical diagnosis.

## 2. Results and Discussions

**Scheme 1:**
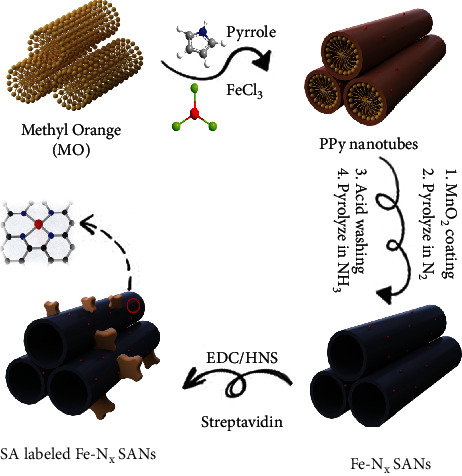
Schematic diagram of preparing SA-labeled Fe-N_x_ SANs.

The synthesis route of SA labeled Fe-N_x_ SANs is shown in Scheme 1. Methyl Orange (MO) micelle soft template was first created in the water phase, and pyrrole monomers and FeCl_3_ were added into the above solutions to form PPy nanotubes. Then, KMnO_4_ was added to form a layer of MnO_2_ coated on the surface of PPy nanotubes in order to produce more single-atom active sites due to nanoconfinement effect. Specifically, MnO_2_ coating can confine atoms into precursors, thus achieving high atomic distribution of Fe iron, reducing aggregation during pyrolysis, and greatly enhancing the number of Fe-N_x_ active sites. Moreover, free migration of iron species could be restricted, further improving its catalytic performance [29, 30]. Subsequently, the Fe-N_x_ SANs were acquired through a typical pyrolysis step under a N_2_ environment followed by an acid leaching process, in which the aggregated Fe atoms and MnO_2_ coating could be removed. Herein, the obtained Fe-N_x_ SANs can catalyze H_2_O_2_ to generate hydroxyl radicals via Fenton reaction, which can be recognized as a superior peroxidase-like activity [31]. Next, the synthesized Fe-N_x_ SANs were treated with N-(3-dimethylamino propyl)-N′-ethylcarbodiimide hydrochloride (EDC) and N-hydroxysuccinimide (NHS), then modified with SA to bind biotinylated A*β* 1-40 antibody, which is proved by the Fourier transform infrared spectra successfully (Figure S1) [32]. Thereinto, the biotin can react with SA-conjugated labels, forming the strongest known noncovalent interaction between a protein and a ligand [33]. Notably, the interaction is rapid and maintains being robust in extreme conditions of pH and temperature. Through these steps, the obtained SA-modified Fe-N_x_ SANs can substitute HRP-streptavidin in the commercial ELISA. Therefore, we utilized these peroxidase-like Fe-N_x_ SANs to develop a new SAN-LISA kit to enhance the detection performance of A*β* 1-40.

**Figure 1:**
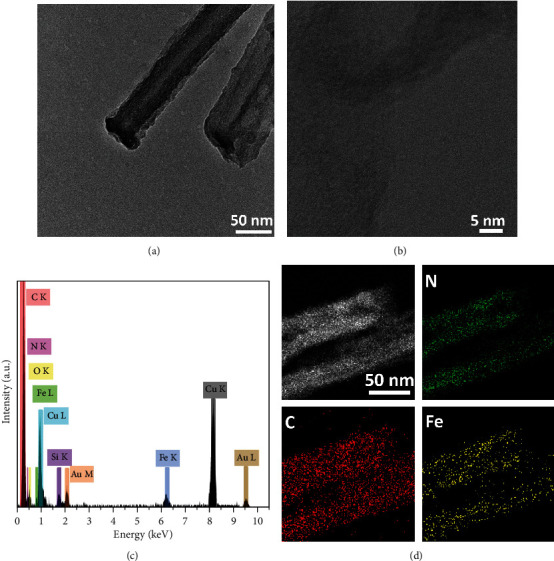
(a) TEM image of Fe-N_x_ SANs. (b) HRTEM image of the Fe-N_x_ SANs. (c) EDS elemental analysis of Fe-N_x_ SANs. (d) STEM image of Fe-N_x_ SANs and EDS elemental mapping results of C, N, and Fe.

The morphologies of PPy nanotube and MnO_2_ coating PPy nanotube were confirmed by Transmission electron microscopy (TEM), shown in Figure S2. In Scheme 1, the well-defined Fe-N_x_ SANs had a typical nanotube structure with a diameter of around 50 nm. Moreover, distorted graphite layers were found in Fe-N_x_ SANs (Figure 1(a)) by high-resolution TEM (HRTEM). This graphite structure could provide enriched defects and nanopores, which would anchor abundant atomic Fe-N_x_ moieties. A N_2_ adsorption/desorption test was carried out to evaluate the detailed textural structure (Figure S3). The Brunauer–Emmett–Teller (BET) surface area of Fe-N_x_ SANs was 648.16 m^2^/g. The large surface area enabled the synthesized Fe-N_x_ SANs to host more Fe–N_x_–C moieties, thus achieving high peroxidase-like activity. Auxiliary energy-dispersive X-ray spectroscopy (EDS) elemental analysis demonstrated that the Fe-N_x_ SANs were comprised of C, N, and Fe (Figure 1(b)). Here, the absence of Mn signal meant that the MnO_2_ coating was removed successfully. The Si, Cu, and Au signals were introduced by TEM technology (such as EDS probes or TEM grid), which was also proved by the X-ray photoelectron spectroscopy (XPS) spectrum (Figure S4). Moreover, the EDS mapping of C, N, and Fe was conducted, as shown in Figure 1(c). All elements were distributed uniformly in the Fe-N_x_ SANs, indicating that the Fe-N_x_ could be incorporated into the PPy matrix. Besides, no Fe clusters were observed, which was because the aggregated Fe species were washed out during acid treatment and the remaining Fe existed as isolated atoms.

**Figure 2:**
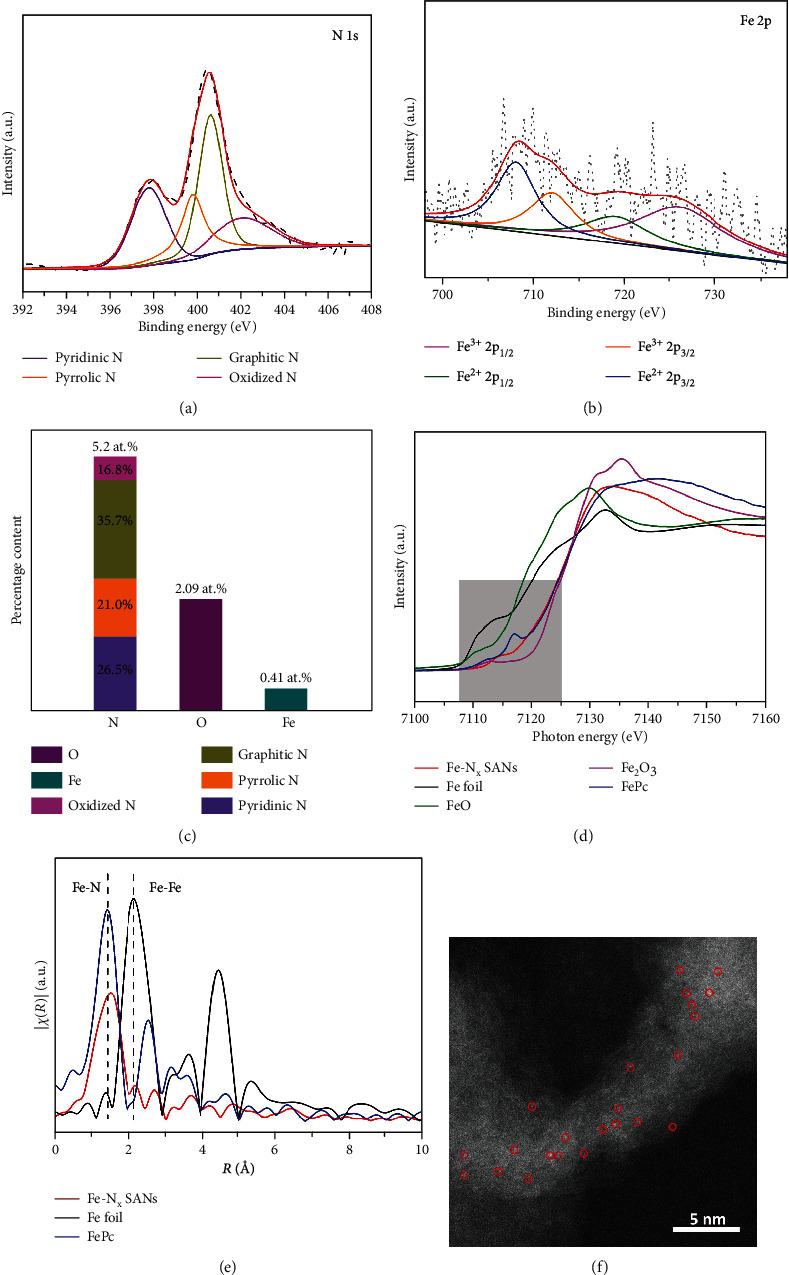
(a, b) High-resolution N 1 s and Fe 2p spectra of Fe-N_x_ SANs, respectively. (c) N, O, and Fe contents in Fe-N_x_ SANs. (d) Fe K-edge XANES spectrum of Fe-N_x_ SANs and reference samples of FePc, Fe foil, FeO, and Fe_2_O_3_. (e) FT *k*^3^-weighted EXAFS spectrum of Fe-N_x_ SANs, FePc, and Fe foil. (f) HAADF-STEM image of Fe-N_x_ SAN sample.

In order to measure the chemical composition of Fe-N_x_ SANs, high-resolution XPS spectra with curve fitting of N 1 s and Fe 2p were adopted, and the results are shown in Figures 2(a) and 2(b). For N 1 s, the spectrum of Fe-N_x_ SANs could be fitted into four peaks at 397.7 eV, 399.7 eV, 400.7 eV, and 402.1 eV, corresponding to Fe-N_x_ or pyridinic N, pyrrolic N, graphitic N, and oxidized N, respectively [34]. Here, we fitted the pyridinic N and Fe-N_x_ in one peak because of the small difference in binding energy between Fe-N_x_ and pyridinic N [35]. For Fe 2p, four peaks of 707.9 eV, 712.1 eV, 718.9 eV, and 723.5 eV and 725.9 eV were assigned to Fe^2+2^p_2/3_, Fe^3+^2p_2/3_, Fe^2+^2p_1/2_, and Fe^3+^2p_1/2_ on the basis of binding energies, respectively [36]. The deconvolution method using Gaussian-Lorentz curve fittings was adopted to conduct the semiquantitative analysis of all the elements [37, 38].  Figure 2(c) shows that the N and Fe contents were 5.02 at.% and 0.41 at.%, respectively, which correspond to previously published works of single-atom Fe-N-C materials [34, 39]. The percentage of defective N configurations (pyridinic and pyrrolic N), regarded as coordination sites for single Fe atoms, was high. Moreover, compared to traditional PPy nanotube-based Fe-N-C materials (0.35 at.% [34]), the nanoconfinement strategy enhanced Fe loading significantly. The Fe K-edge X-ray absorption near-edge structure (XANES) spectra (Figure 2(d)) of Fe-N_x_ SANs and reference samples of iron (II) phthalocyanine FePc, Fe foil, FeO, and Fe_2_O_3_ were obtained. Obviously, the near-edge absorption energy of Fe-N_x_ SANs was located between standard bi- (FeO) and trivalent (Fe_2_O_3_) iron, illustrating that +2 and+3 coexisted in Fe-N_x_ SANs, consistent with XPS results (Figure 2(b)). The Fourier-transform EXAFS curve of Fe-N_x_ SANs in Figure 2(e) showed the Fe-N peak at 1.4 Å and no Fe-Fe peak at 2.1 Å was observed. Moreover, from the K-edge EXAFS oscillations, the spectrum of Fe-N_x_ SANs was distinct from those of Fe foil and Fe oxides, but almost the same as that of Fe single-atom reference FePc (Figure S5b), which could further demonstrate that Fe was atomically dispersed in Fe-N_x_ SANs. Such a structure is also similar to natural HRP (Figure S5a), thereby possessing intrinsic peroxidase activity.

Subsequently, in order to confirm the distribution of Fe species in Fe-N_x_ SANs at atomic levels, aberration-corrected scanning TEM (STEM) characterizations were carried out (Figure 2(f)). It clearly showed that the Fe species were uniformly dispersed into the PPy matrix and formed single-atom Fe sites, which were the bright dots circled with red marks. In addition, no nanoparticles were observed at the atomic level, which again proved that no aggregated Fe species existed in Fe-N_x_ SANs. All the results illustrated that enriched atomic Fe-N_x_ moieties had been doped in the PPy matrix effectively.

**Figure 3:**
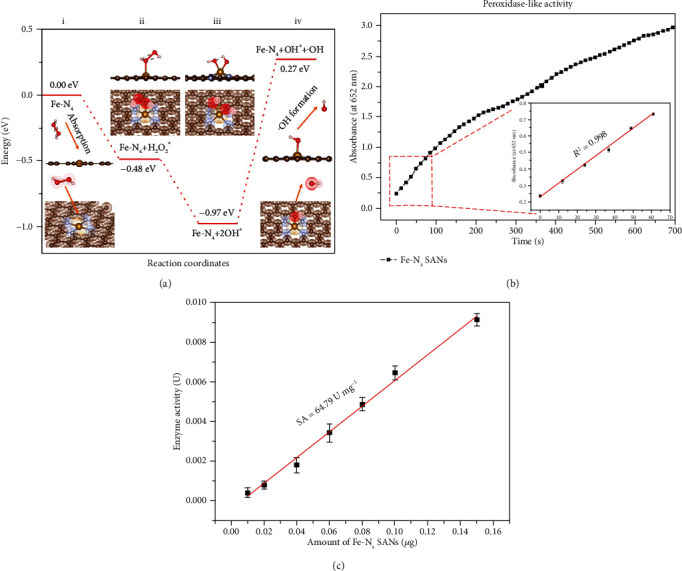
(a) The free energy diagrams of the Fe-N_4_ model in catalytic process. (b) Absorbance-time curve of TMB chromogenic reaction catalyzed by Fe-N_x_ SANs and the corresponding magnified initial linear portion. (c) Specific activities of Fe-N_x_ SANs.

To elucidate the possible peroxidase-like catalytic property of Fe-N_x_ SANs, we performed density functional theory (DFT) calculations to investigate the reaction process of the generation of hydroxyl radicals through catalyzing H_2_O_2_ with the Fe-N_4_ SAN model. As shown in Figure 3(a), the H_2_O_2_ molecule is firstly adsorbed on the Fe active site in the Fe-N_4_ SAN with an adsorption energy of -0.48 eV. Then, the H_2_O_2_ molecule easily dissociates and then a hydroxyl group desorbs from the adsorbed site, resulting in the generation of an active hydroxyl radical and adsorption of a hydroxyl group at the Fe-N_4_ active site. The energy diagram also matched previous reports [40, 41], and the calculated reaction energy from the initial step to the final step was 0.27 eV. Such low reaction energy confirmed the potential peroxidase-like catalytic property of Fe-N_x_ SANs. Further specific peroxidase-like activities and steady-state kinetics properties of Fe-N_x_ SANs were assessed in acetate buffer (pH = 3.6). Thereinto, 3,3′,5,5′-tetramethylbenzidine (TMB) was employed as the allochroic substrates. First, the TMB chromogenic reaction curve of absorbance to time was obtained, and the sample without adding H_2_O_2_ was served as a reference. The result is shown in Figure 3(b). It is clear that the absorbance at 652 nm increased with reaction time and the absorbance to reaction time was linear in the first minute with *R*^2^ coefficient close to 1 in linear regression analysis. The catalytic activity of Fe-N_x_ SANs expressed in units (*U*) was further assessed. Specifically, different amounts of Fe-N_x_ SANs were used to trigger chromogenic reaction of TMB. The first 60s was chosen as an initial time, and the results are in Figure 3(c). The peroxidase-mimic activity of the Fe-N_x_ SANs was calculated to be 64.79 U mg^−1^, which is higher than that of the reported Fe-N_x_/SAN and conventional nanozymes (Table S1, Supporting information), further proving that the synthesized SANs possessed unprecedented peroxidase-like properties. This is due to the reason that those active sites of Fe-N_x_ have similar effective structures to natural enzymes. What is more, owing to single-atom Fe, the atom utilization could become 100% theoretically. In other words, every single atom can work as an active site to catalyze H_2_O_2_.

**Figure 4:**
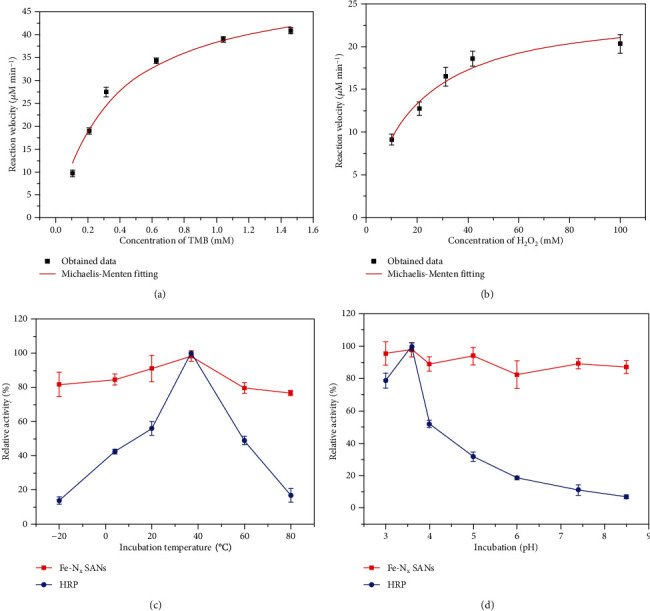
(a, b) Steady-state kinetics curves of Fe-N_x_ SANs toward TMB and H_2_O_2_, respectively; (c, d) Robustness of Fe-N_x_ SANs against the harsh environment of temperature and pH, respectively.

Then, the kinetics of peroxidase-mimicking catalysis of Fe-N_x_ SANs was analyzed, as shown in Figures 4(a) and 4(b). The steady-state kinetics curves of Fe-N_x_ SANs towards TMB substrates and H_2_O_2_ were obtained. Moreover, Michaelis constants (*K*_*m*_) of the steady-state kinetics were obtained by fitting in the Michaelis-Menten model and compared with that of HRP. The *K*_*m*_ of Fe-N_x_ SANs with TMB and H_2_O_2_ as the substrate is slightly lower than that of HRP, demonstrating that the synthesized SAN has a comparable affinity of HRP. Besides, the steady-state kinetics curves of HRP toward H_2_O_2_ and TMB are shown in Figure S6; *K*_*cat*_ and *K*_*cat*_/*K*_*m*_ of Fe-N_x_ SANs and HRP were calculated and listed in Table S2, again indicating the excellent catalytic performance of Fe-N_x_ SANs. Also, the stability of Fe-N_x_ SANs in harsh environments was evaluated, as shown in Figures 4(c) and 4(d). The curve demonstrated that the SANs maintained excellent stability with pH and temperature variation, while HRP gradually lost its activities when pH was higher than four or the temperature was not close to 40°C. These results indicate that the Fe-N_x_ SANs can retain much better robustness in harsh environments.

**Figure 5:**
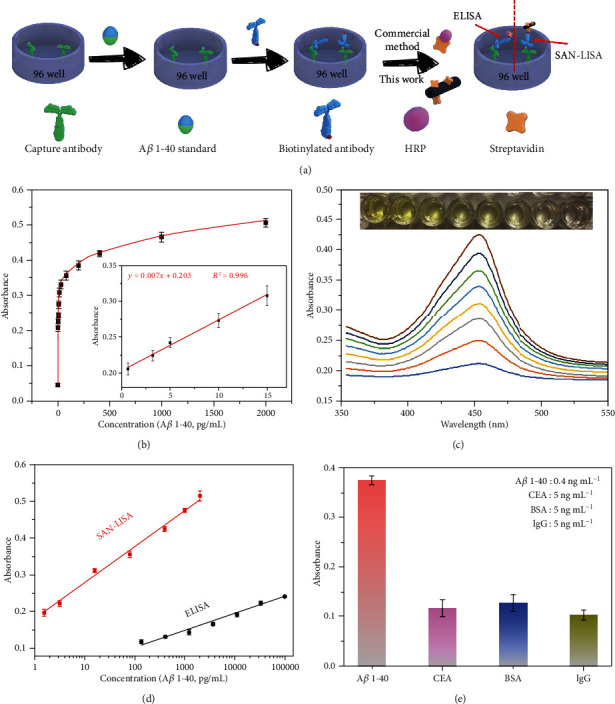
(a) Schematic illustration of SAN-LISA for the detection of A*β* 1-40. (b) The curve of SAN-LISA for the detection of A*β* 1-40 ranging from 1 pg/mL to 2000 pg/mL. (c) Absorbance spectra of various concentrations of A*β* 1-40 detected by SAN-LISA. (d) Standard curves of SAN-LISA (A*β* 1-40 ranging from 1 to 2000 pg/mL) and ELISA (A*β* 1-40 ranging from 100 pg/mL to 100 ng/mL). (e) Specificity of SAN-LISA (A*β* 1-40 of 400 pg/mL; CEA, BSA and IgG of 5 ng/mL, respectively).

Amyloid beta 1-40 is a typical biomarker of detecting Alzheimer's disease (AD). However, the concentration of A*β* 1-40 is in pg/mL to ng/mL level in human serum which requires sensitive and accurate detection in the early diagnosis of AD. Herein, a typical sandwich-type SAN-linked immunosorbent assay (SAN-LISA) was built to detect A*β* 1-40, shown in Figure 5(a). The curve of SANs detecting A*β* 1-40 was obtained and is shown in Figure 5(b). The linear range was 1 pg/mL to 2000 pg/mL. The low concentration range between 0 and 15 pg/mL is shown in the inserted figure in Figure 5(b). By applying to the equation 3*S*/*K*, where *S* and *K* referred to the standard deviation of blank sample and slope of the standard curve, respectively, the limit of detection (LOD) was calculated to be 0.88 pg/mL, which is low enough to meet the detection requirement of human serum. Absorbance spectra of various concentrations of A*β* 1-40 detected by SAN-LISA and their corresponding colorimetric signal are shown in Figure 5(c). It was shown clearly that the signal intensities increased with elevated concentrations of A*β* 1-40. As comparison, the calibration curve of commercial ELISA for the A*β* 1-40 detection was analyzed and is shown in Figure S7. By applying the previous equation, the LOD of the traditional ELISA was calculated to be 9.98 pg/mL, which was more than ten times higher than that of SAN-LISA. The enhanced sensitivity was due to the ultrahigh surface area which could hold more active sites. Furthermore, we evaluated the sensitivity of SAN-LISA by comparing the signal between traditional ELISA and proposed SAN-LISA (Figure 5(d)). The results proved that SAN-LISA has better sensitivity, with much higher absorbance on much lower concertation of A*β* 1-40. What is more, we further studied the detection performance of the two methods with the same A*β* 1-40 concentrations to further prove the satisfactory detection sensitivity of SAN-LISA, shown in Figure S8. As shown in Table 1, compared with previously reported detection results of A*β* 1-40 using different methods, the proposed SAN-LISA method exhibits superior detection performance. Lastly, the specificity of SAN-LISA was analyzed, as displayed in Figure 5(e). A*β* 1-40 exhibited a distinct signal, while the other competing proteins had negligible signals, indicating the satisfactory specificity of SAN-LISA.

## 3. Conclusion

In summary, we have successfully synthesize a Fe-N_x_ single-atom catalyst with outstanding peroxidase-mimicking activity, which is mainly attributed to the ultra-large surface area of carbon support that forms more active sites and enables 100% Fe atom utilization. It also shows excellent robustness in harsh environments. Most importantly, novel Fe-N_x_ SAN-LISA has been developed to enhance the detection performance of A*β* 1-40, exhibiting a sensitivity with LOD of 0.88 pg/mL. This result is much lower than that of the commercial ELISA kit (9.98 pg/mL), which meets the requirement of effective detection of A*β* 1-40. Based on the high activity of Fe-N_x_ SANs and improved ELISA performance, the peroxidase-like SANs show great potential and pave a new way to design ELISA kits with improved sensitivity for detecting various target biomarkers.

## 4. Experimental Section

### 4.1. Preparation of Fe-N_x_ SANs

500 mg of MO was dissolved in DI water; then, 5 g of FeCl_3_ and 1.5 mL pyrrole were added under vigorous stirring to form a Fe^3+^-doped PPy nanotube. MnO_2_-coated PPy nanotubes were prepared by dispersing a certain amount of KMnO_4_ into the aforementioned solution. The product was pyrolyzed at 900°C under the nitrogen atmosphere, and the MnO_2_ coating could be removed by leaching for 8 h with 5% H_2_SO_4_ (*v*/*v*) [48]. Finally, the Fe-N_x_ SANs were obtained after the second heat treatment at 900°C under ammonia.

### 4.2. Fabrication of SA-Labeled Fe-N_x_ SANs

First of all, the tubed Fe-N_x_ SANs were shattered under vigorous sonication and dispersed in PBS (0.5 mg/ml), then adjusted by K_2_CO_3_ to reach pH = 6.0 and ultrasonicated for 1 h. Secondly, the solution was activated by N-(3-dimethylaminopropyl)-N′-ethylcarbodiimide (EDC: 2 mg/mL) and N-hydroxysuccinimide (NHS: 4 mg/mL) under shaking for 30 minutes, and then, the mixture was centrifuged and washed three times to form the activated Fe-N_x_ SANs. SA (100 *μ*g/ml in PBS) was incubated with activated Fe-N_x_ SANs at 37°C for 1 hour, and the mixture was centrifuged for three times to remove unbonded SA. Lastly, the products were passivated with 1% BSA for 30 minutes and dispersed in 1 mL of PBS. Herein, the SA-labeled Fe-N_x_ SANs were broken down to nanosize via an intense ultrasound treatment before further using in ELISA.

### 4.3. Detection of Amyloid Beta 1-40 by SAN-Linked Immunosorbent Assay (SAN-LISA)

Schematic illustration of the procedures to determine the level of amyloid beta 1-40 through the ultrasensitive ELISA method employing the Fe-N_x_ SANs is shown in Figure 5(a). Firstly, different amounts of amyloid beta 1-40 standard were added into a 96-well plate and incubated at 37°C for 2.5 h. Each well was washed three times, and then, 200 *μ*L of PBST (PBS containing 0.5 wt % of TWEEN-20) containing 1 wt % BSA was added into it to block the unbonded primary antibody at 37°C for 1.5 h. Secondly, 100 *μ*L of the prepared biotinylated amyloid beta 1-40 was added to each well and incubated for 1 h with gentle shaking, then the plate was washed with wash buffer for three times. Thirdly, 50 *μ*L of SA labeled Fe-N_x_ SANs was added into each well and shaken for 45 min. Finally, a chromogenic reaction was conducted. Specifically, 100 *μ*L TMB was added to each well and the mixture was incubated for 10 min at room temperature under gentle shaking. Then, 50 *μ*L stop solution was added to stop the reaction and absorbance data were collected at 450 nm immediately upon color change.

## Figures and Tables

**Table 1:** Reviews of the detection of A*β* 1-40 with different methods.

Techniques	LOD (pg mL^−1^)	Linear range (pg mL^−1^)	Reference
SAN-LISA	0.88	1-2000	This work
Electrochemical immunoassay	19	20-12500	[42]
SWV∗ at GCE∗	7 × 10^5##^	Nonlinear	[43]
Microfluidic droplet	2165^#^	NP	[44]
EIS∗	2468^#^	43.3-4.33 × 10^5#^	[45]
SPR∗	86.6^###^	86.6-865.9^#^	[46]
SWV∗	8.6 × 10^5#^	1.772 × 10^6^-8.66 × 10^6#^	[47]

SWV: Square Wave Voltammetry; GCE: Glassy Carbon Electrode; SPR: Surface Plasmon Resonance; ECL: Electrochemiluminescence (ECL) immunosensor; EIS: Electrochemical Impedance Spectroscopy. ^#^Value was expressed in nM and converted to pg mL^−1^. ^##^Value was expressed in *μ*g mL^−1^ and converted to pg mL^−1^. ^###^Value was expressed in pM and converted to pg mL^−1^.

## Supplementary Materials

Supplementary Materials Figure S1: the Fourier transform infrared spectra of the Fe-N_x_ SANs and SA-labeled Fe-N_x_ SANs. The strong peak at 1638 cm^−1^ which corresponds to the amide I shows that streptavidin is already successful labeled on Fe-N_x_ SANs. Figure S2: morphology of PPy nanotube and MnO_2_-coated PPy nanotube. Figure S3: N2 adsorption-desorption isotherm of Fe-N_x_ SANs. Figure S4: X-ray photoelectron spectroscopy (XPS) spectrum of Fe-N_x_ SANs. Figure S5: structure of natural (a) HRP and (b) iron (II) phthalocyanine (FePc). Figure S6: steady-state kinetics curves of HRP toward (a) H_2_O_2_ and (b) TMB. Figure S7: standard curve of commercial EISA for the detection of A*β* 1-40 (A*β* 1-40 ranging from 0.1 to 100 ng/mL) and its linear range. Figure S8: standard curve of commercial ELISA and SAN-LISA (A*β* 1-40 ranging from 100 pg/mL to 10 ng/mL). Table S1: comparison of peroxidase-like specific activity (U/mg) of Fe-N_x_, other published nanozymes, and natural HRP. Table S2: comparison of steady-state kinetics parameters of Fe-N_x_ SANs and natural HRP.
