# Supplementary material for: Single-Atom Nanozymes Linked Immunosorbent Assay for Sensitive Detection of Aβ 1-40: A Biomarker of Alzheimer's Disease
Source: Research (Wash D C). 2020 Oct 19;2020:4724505. doi: 10.34133/2020/4724505 (PMC7592081; doi:10.34133/2020/4724505)
Supplement: Supplementary Materials — Figure S1: the Fourier transform infrared spectra of the Fe-Nx SANs and SA-labeled Fe-Nx SANs. The strong peak at 1638 cm−1 which corresponds to the amide I shows that streptavidin is already successful labeled on Fe-Nx SANs. Figure S2: morphology of PPy nanotube and MnO2-coated PPy nanotube. Figure S3: N2 adsorption-desorption isotherm of Fe-Nx SANs. Figure S4: X-ray photoelectron spectroscopy (XPS) spectrum of Fe-Nx SANs. Figure S5: structure of natural (a) HRP and (b) iron (II) phthalocyanine (FePc). Figure S6: steady-state kinetics curves of HRP toward (a) H2O2 and (b) TMB. Figure S7: standard curve of commercial EISA for the detection of Aβ 1-40 (Aβ 1-40 ranging from 0.1 to 100 ng/mL) and its linear range. Figure S8: standard curve of commercial ELISA and SAN-LISA (Aβ 1-40 ranging from 100 pg/mL to 10 ng/mL). Table S1: comparison of peroxidase-like specific activity (U/mg) of Fe-Nx, other published nanozymes, and natural HRP. Table S2: comparison of steady-state kinetics parameters of Fe-Nx SANs and natural HRP. [file 4724505.f1.docx]

**Single Atom Nanozymes linked Immunosorbent Assay for Sensitive detection of A*β* 1-40, a Biomarker of Alzheimer’s Disease**

**Zhaoyuan Lyu,^1^ Shichao Ding,^1^ Nan Zhang,^2^ Yang Zhou,^1^ Nan Cheng,^1^ Maoyu Wang,^3^ Mingjie Xu,^4^ Zhenxing Feng,^3^ Xiangheng Niu,^1^ Yuan Cheng,^2^ Chao Zhang,^5^ Dan Du,^1^ Yuehe Lin^1^**

^1^ School of Mechanical and Materials Engineering, Washington State University, Pullman, WA, 99164, USA

^2^ Institute of High Performance Computing, Institute of High Performance Computing, 138632, Singapore

^3^ School of Chemical, Biological, and Environmental Engineering, Oregon State University, Corvallis, OR, 97331 USA

^4^ Irvine Materials Research Institute (IMRI), University of California, Irvine, CA, 92697, USA

^5^ Department of Electrical and Computer Engineering, National University of Singapore, Engineering Drive 3, 117583 Singapore

Correspondence should be addressed to Dan Du and Yuehe Lin; [annie.du@wsu.edu](mailto:annie.du@wsu.edu); yuehe.lin@wsu.edu;

**Materials**

Methyl Orange (MO) and Pyrrole (reagent grade, 98%) were obtained from Sigma-Aldrich, USA; Iron(iii) chloride (anhydrous, 98%) was provided by Thermo Fisher Scientific, USA; Potassium permanganate was supplied by Fisher Scientific, USA. 3,3′,5,5′-tetramethylbenzidine (TMB), hydrogen peroxide (H_2_O_2_), bovine serum albumin (BSA), N-(3-dimethylamino propyl)-N′-ethylcarbodiimide hydrochloride (EDC) and N-hydroxysuccinimide (NHS), Streptavidin (SA) and dimethyl sulfoxide were purchased from Sigma, USA. Amyloid beta 1-40 Human ELISA kit was gotten from Abcam, USA.

**Instruments and characterization**

Images of materials were obtained by TEM (Tecnai G2 T20, 200 kV; JEOM Grand ARM300F, 300 kV); elemental analysis was conducted by X-ray photoelectron spectroscopy (XPS, Escalab 250, Al Kα). The X-ray absorption spectroscopy measurement at Fe K-edge was performed at the Advanced Photon Source (APS) on the bending-magnet beamline 9-BM-B with electron energy cof 7 GeV and average current of 100 mA. The radiation was monochromatized by a Si (111) double-crystal monochromator. All absorption spectra and fluorescence spectra were performed by Tecan Safire2 Multi-Mode Microplate Reader. The specific surface area of the sample was investigated with an automatic volumetric sorption analyzer (ASAP 20209 M) which N_2_ acts as the adsorbate at -196^o^C.

**Evaluation of the Peroxidase-like properties of Fe-N_x_ SANs**

Peroxidase-like properties of Fe-N_x_ SANs were conducted following standard protocol (Nature protocols, 2018, 13(7): 1506). Specifically, TMB was used as a substrate to verify the peroxidase-like feature of Fe-N_x_ SANs. In a typical measurement, Fe-N_x_ SANs was dispersed in HAc-NaAc buffer with PH = 3.6 and distributed into a 96-well plate. Then 100 uL TMB (10 mg/mL in DMSO) was added. The mixture was incubated under 37^o^C in dark for 1min, then H_2_O_2_ was added to a final concertation of 1 M. The reaction-time curve of Fe-N_x_ SANs was plotted using the absorbance at 652 nm against the reaction time. The catalytic activity units (U) was evaluate by detecting the absorbance at 652 nm immediately and recorded at a 10 s interval within 700 s. After subtracting the background, the nanozyme activity expressed in units (U) was calculated according to the following equation:

$$b_{\mathrm{nanozyme}}= \frac{V}{\varepsilon l} \times\frac{\Delta A}{\Delta t}$$

In which *b*_nanozyme_ refers to the nanozyme activity (U), V is volume of the reaction solution (μL), *ε* is the molar absorption coefficient of TMB substrate (39,000 M^-1^ cm^-1^ at 652 nm), *l* is the optical path length through reaction solution (cm) and Δ*A*/Δ*t* is the initial rate (within 1 min) of the absorbance change (min^-1^).

When using different amounts of Fe-N_x_ SANs to measure the peroxidase-like activity, the specific nanozyme activity was determined by the following equation:

$$a_{\mathrm{nanozyme}}= \frac{b_{\mathrm{nanozyme}}}{m}$$

where *a*_nanozyme_ is the specific activity of nanozyme (U mg^-1^) and *m* is the nanozyme amount (mg).

For the steady-state kinetic measurements of peroxidase-like Fe-N_x_ SANs, 10 μL TMB solution with different concentrations (from 0 to 3.5 mM) and a certain volume of 1 M H_2_O_2_ solution were added to NaAc-HAc buffer (pH 3.6) to a ﬁnal concentration of 1 M. After 50 μL of 1 μg/mL Fe-N_x_ SANs solution was added and mixed together for reaction, the absorbance at 652 nm was immediately recorded at a 10 s interval within 60 s. Then, the initial rates of the chromogenic reaction upon different TMB concentrations were obtained. The substrate concentration-dependent reaction rate curves were fitted with Michaelis-Menten model and Michaelis constant *K_m_* and *K_cat_* were calculated according the following Michaelis-Menten equation:

$$v = \frac{v_{\max}[S]}{K_{m}+ [S]} K_{cat}=\frac{\upsilon_{max}}{[E]}$$

where *v* is the initial rate of the chromogenic reaction, [S] is the TMB concentration and [E] is the nanozyme concentration (M).

Finally, peroxidase-like activity of Fe-N_x_ SANs was carefully analyzed and the steady-state kinetics properties of Fe-N_x_ SANs were also systematically evaluated and compared with HRP.

**DFT computational details**

Vienna ab initio simulation package (VASP) was conducted to all calculations with the with plane-wave pseudopotential method. Generalized gradient approximation (GGA) was utilized to describe the electronic exchange and related effects with Perdew-Burke-Ernzerhof (PBE) functions, while all-potential projector augmented wave (PAW) method was performed to describe the core electrons. Plane wave expansion utilized an energy cutoff of 600 eV, and the force on the relaxed atom was less than -0.03 eV/Å. The Van der Waals (VdW) corrections of DFT-D3 and spin-polarization were considered in all calculations. To study the structure and catalytic mechanism, Fe-N_4_ sites embedded in a periodic (6 × 6) graphene supercell model were established, in which the vacuum region between the periodic plates was 20Å. The Brillouin zone was sampled using a (3 × 3 × 1) k-point grid generated by the Gamma scheme.

The adsorption energy (*E*) were calculated according to

*E* = *E*PGM-SACs - (*E*SACs+ *E*PGM)

where *E*PGM, *E*SACs, and *E*gas-SACs represent the energies of the produced gas molecule, the clean Fe-doped carbon surface, and the corresponding adsorbed gas molecule on Fe-doped carbon surface, respectively.


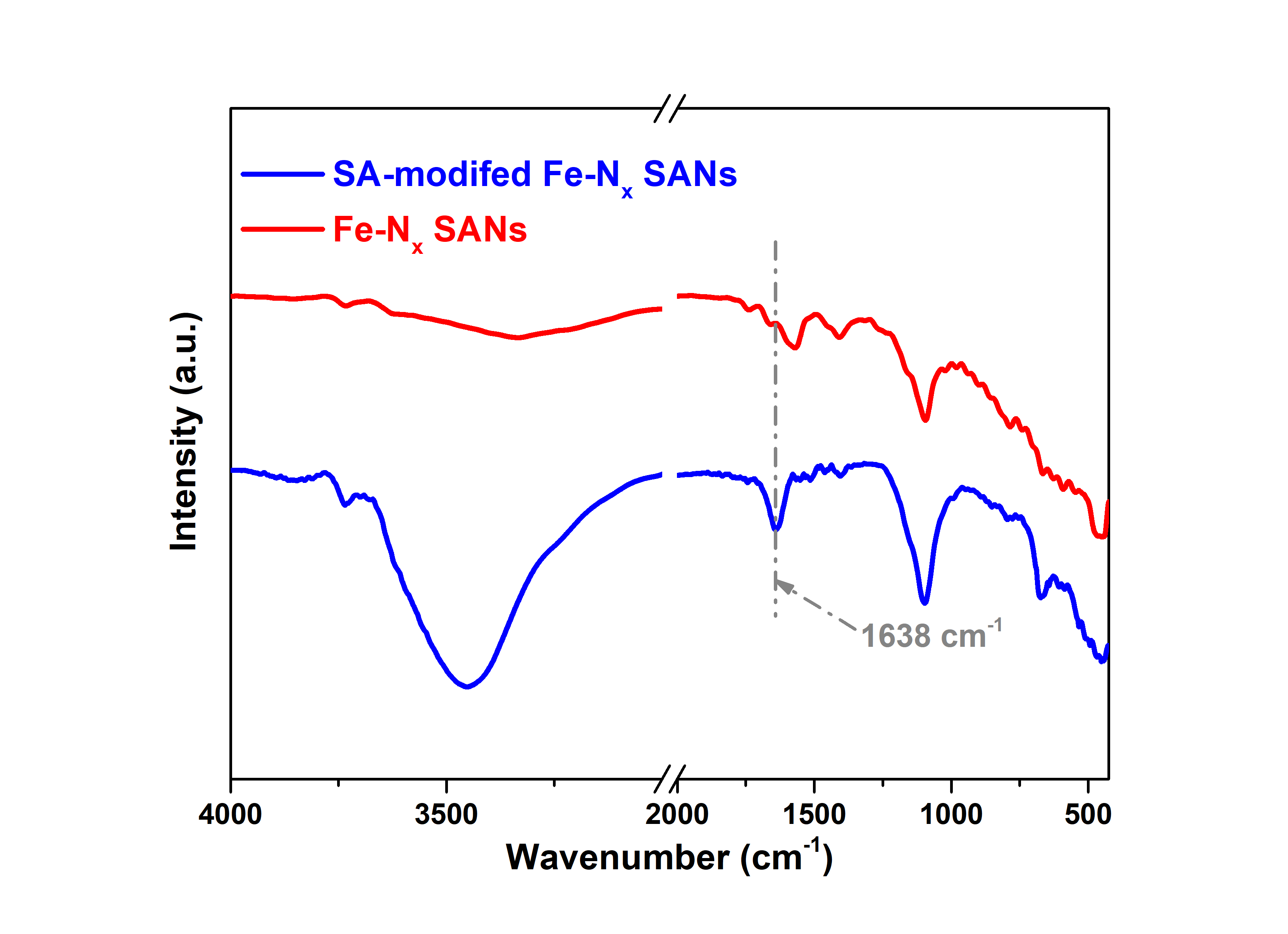


**Figure S1** The Fourier transform infrared spectra of the Fe-N_x_ SANs and SA-labeled Fe-N_x_ SANs The strong peak at 1638 cm^-1^ which corresponds to the amide I show that streptavidin is already successful labeled on Fe-N_x_ SANs.


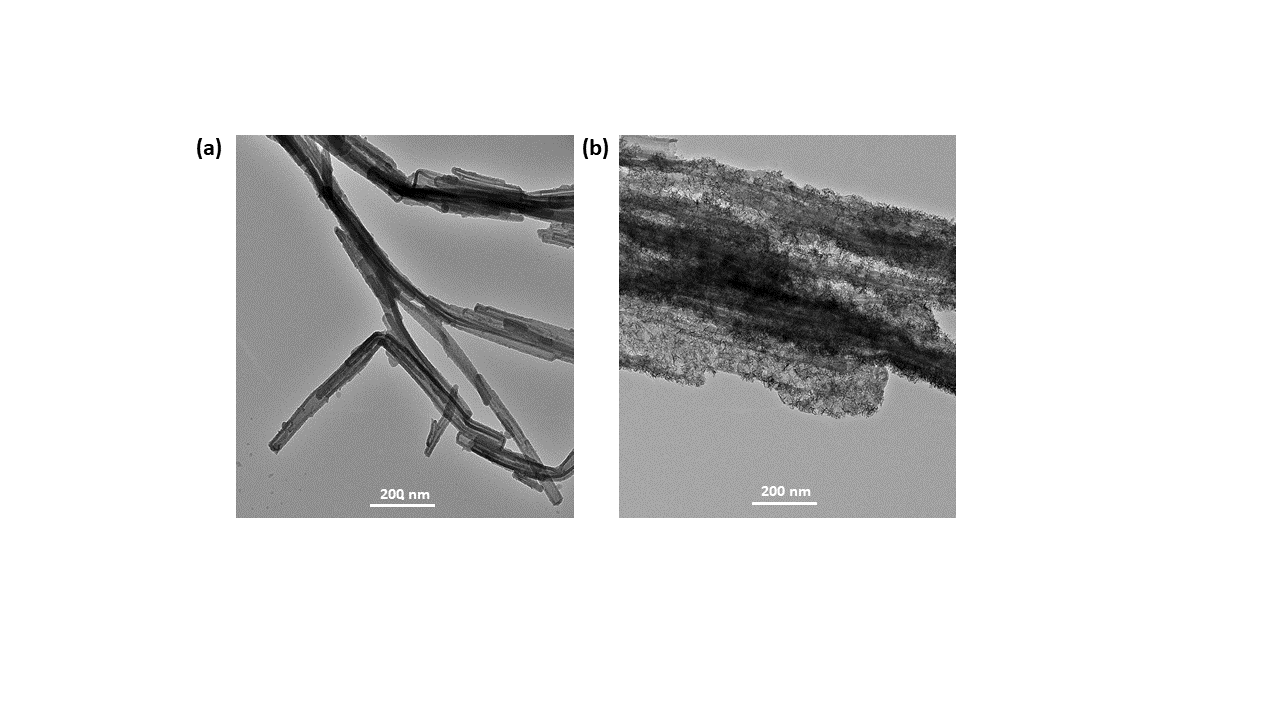


**Figure S2** Morphology of PPy nanotube and MnO_2_ coated PPy nanotube


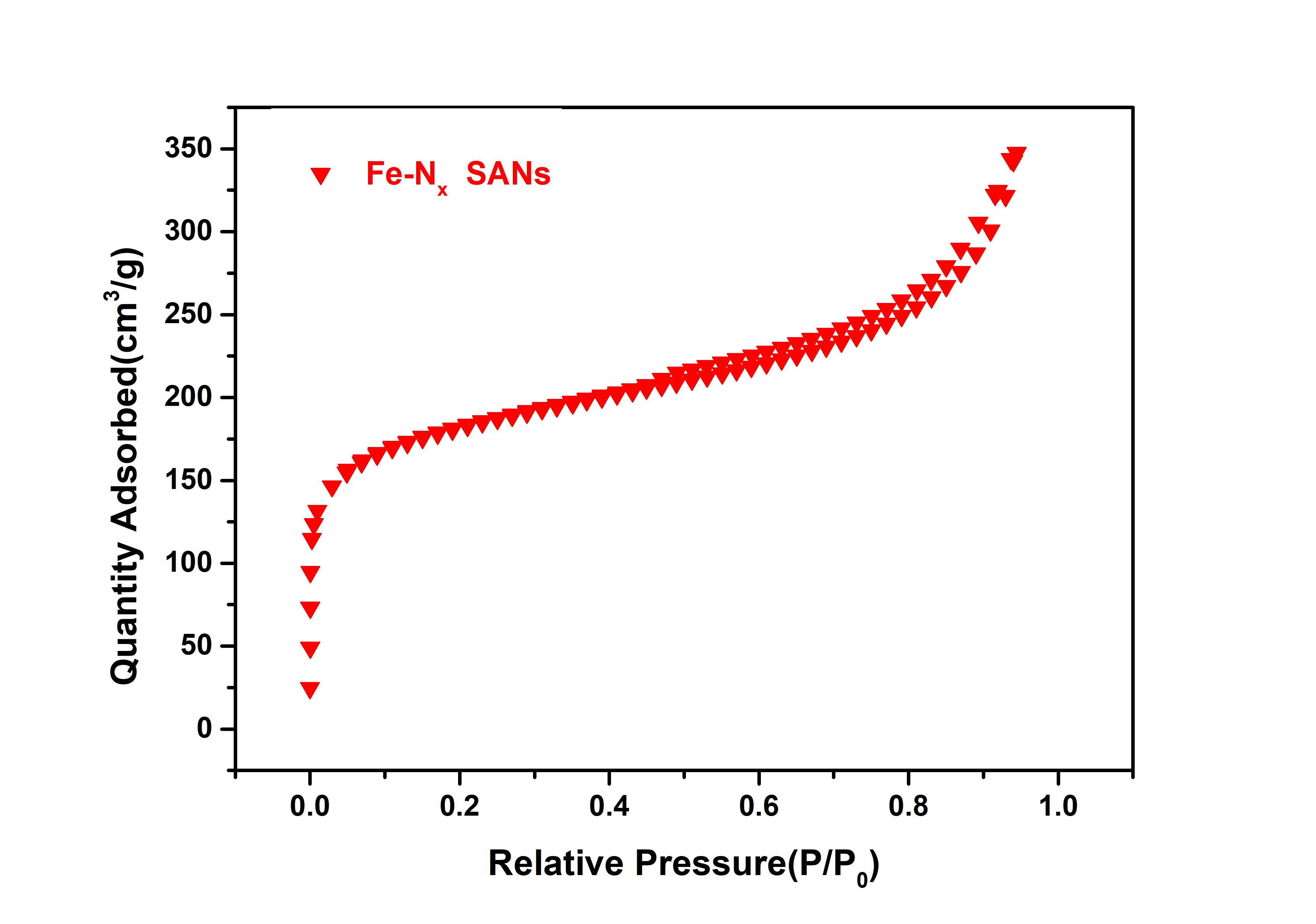


**Figure S3** N_2_ adsorption-desorption isotherm of Fe-N_x_ SANs


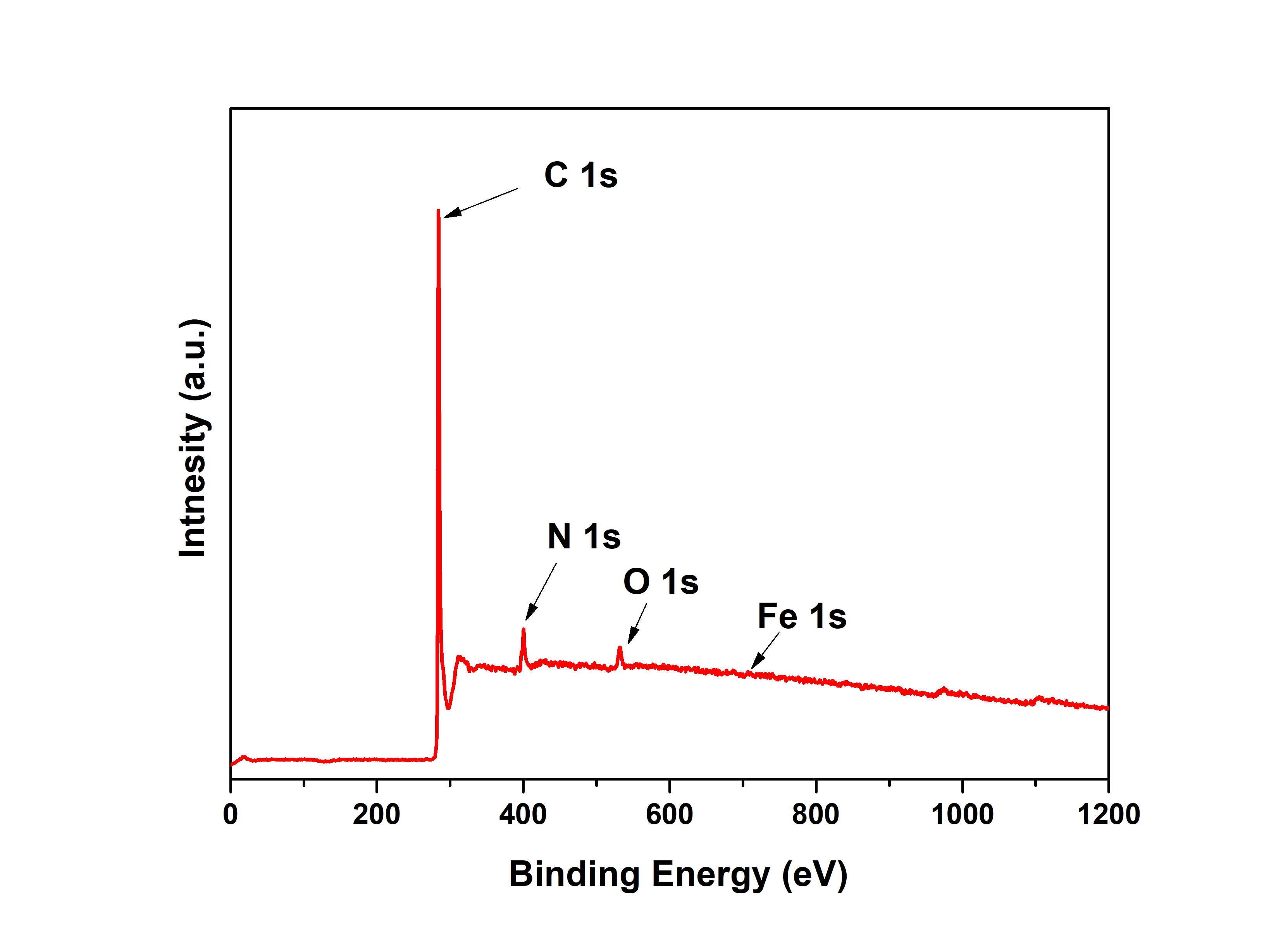


**Figure S4** X-ray photoelectron spectroscopy (XPS) spectrum of Fe-N_x_ SANs


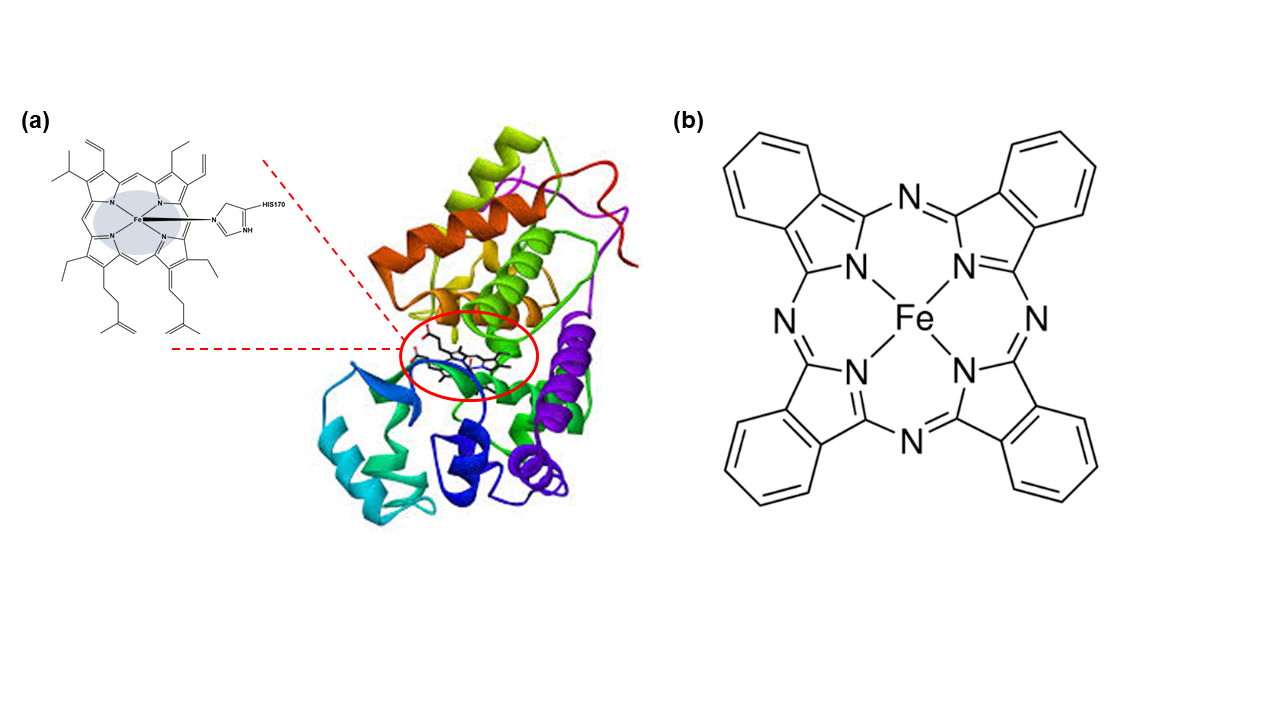


**Figure S5** Structure of natural (a) HRP and (b) iron (II) phthalocyanine (FePc)

**
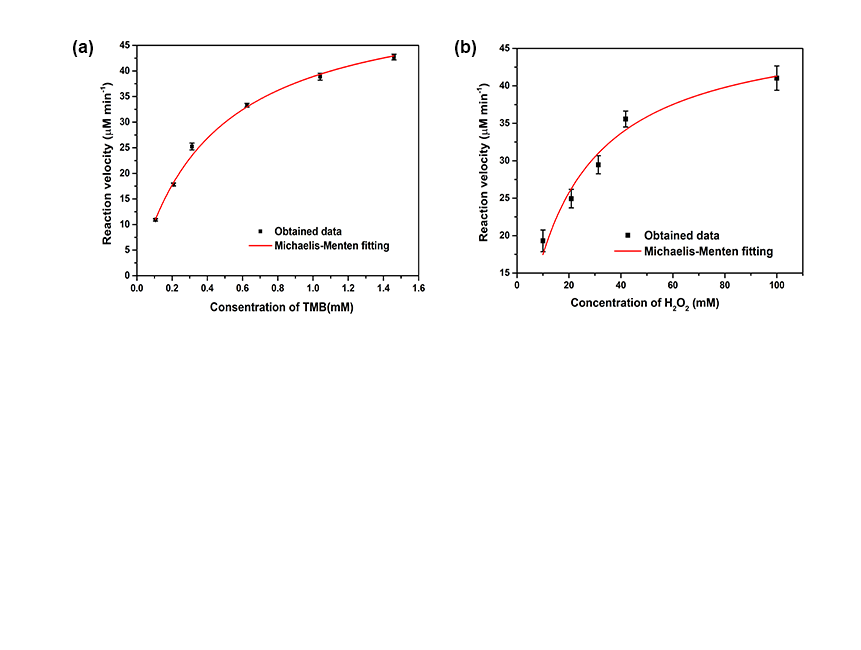
**

**Figure S6** Steady-state kinetics curves of HRP toward (a) H_2_O_2_ and (b) TMB

**
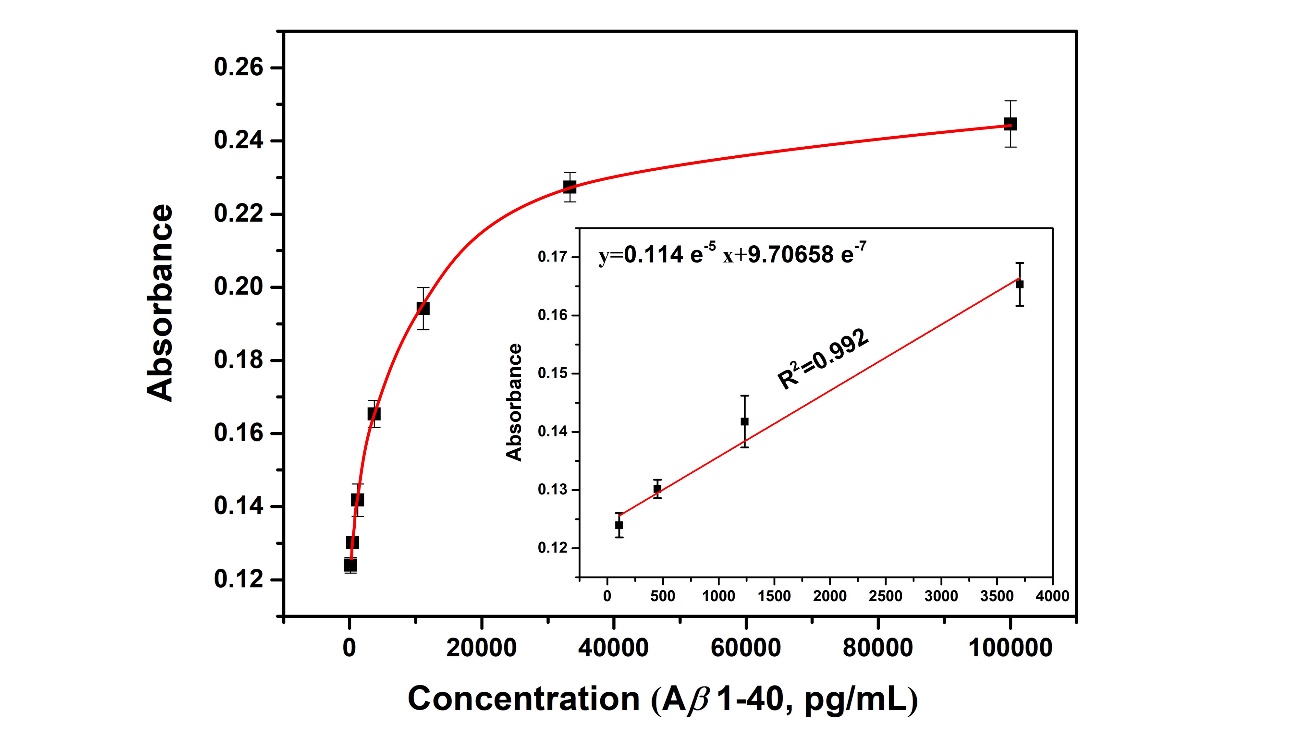
**

**Figure S7** Standard curve of commercial EISA for the detection of A*β* 1-40 (A*β* 1-40 ranging from 0.1 to 100 ng/mL) and its linear range


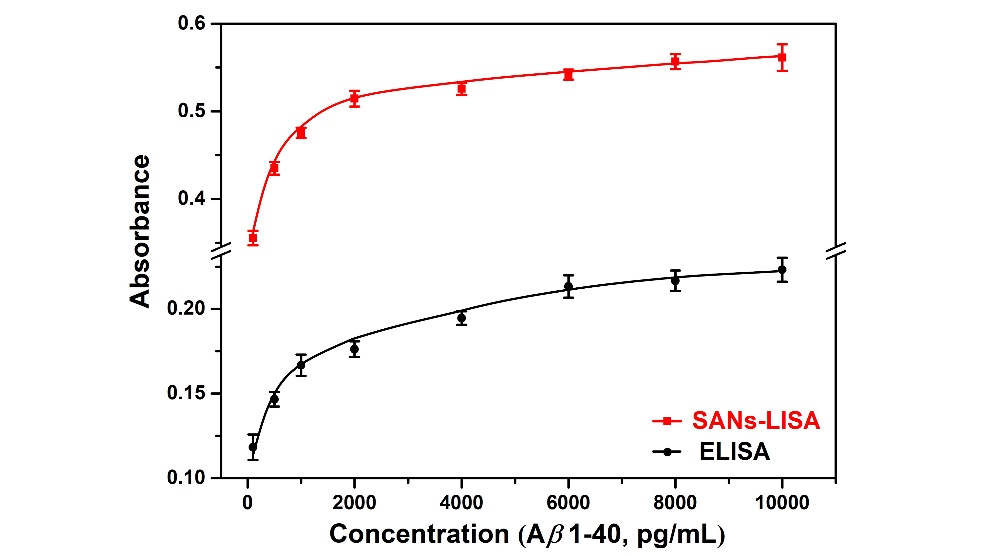


Figure S8 Standard curve of commercial ELISA and SANs-LISA (A*β* 1-40 ranging from 100 pg/mL to 10 ng/mL)

**Table S1.** Comparison of peroxidase-like specific activity (U/mg) of Fe-N_x_, other published nanozymes and natural HRP

| **Enzyme** | **Peroxidase-like specific activity (U/mg)** | **Ref.** |
| --- | --- | --- |
| Fe-N_x_ SANs | 64.79 | This work |
| Fe-N_x_/SAN | 57.76 | *Biosensors and Bioelectronics*, 2019, 142: 111495. |
| Fe SAEs | 6.75 | *Chemical Communications,* 2019, 55(16): 2285. |
| Fe-MOF | 5.086 | *Analytical chemistry,* 2019, 91(21): 13847. |
| Go/Fe-MOF | 7.689 | *Analytical chemistry,* 2019, 91(21): 13847. |
| Fe_3_O_4_ NPs | 5.143 | *Nature protocols*, 2018, 13(7): 1506. |
| Carbon NPs | 3.302 | *Nature protocols*, 2018, 13(7): 1506. |
| Au NPs | 1.633 | *Nature protocols*, 2018, 13(7): 1506. |
| Natural HRP | 297 | *Biosensors and Bioelectronics*, 2019, 142: 111495. |

**Table S2**. Comparison of steady-state kinetics parameters of Fe-N_x_ SANs and natural HRP.

| **Materials** | **[*E*]**  **(M)** | **Substrate** | ***K*_m_**  **(mM)** | ***v*_max_**  **(μM min^-1^)** | ***K*_cat_**  **(min^-1^)** | ***K*_cat_/*K*_m_**  **(M^-1^ min^-1^)** |
| --- | --- | --- | --- | --- | --- | --- |
| Fe-N_x_ SANs | 7.3×10^-11^ | H_2_O_2_ | 17.12 | 24.48 | 3.35×10^5^ | 19.57×10^6^ |
|  |  | TMB | 0.3322 | 51.4 | 7.04×10^5^ | 21.19×10^8^ |
| Natural HRP | 2.5×10^-11^ | H_2_O_2_ | 18.64 | 48.6 | 1.99×10^6^ | 10.6×10^7^ |
|  |  | TMB | 0.4269 | 55.49 | 2.22×10^6^ | 5.2×10^9^ |
